# Digital stress perception and associations with work- and health-related outcomes among general practitioners in Germany: a quantitative study

**DOI:** 10.1186/s12913-025-12653-5

**Published:** 2025-04-11

**Authors:** Annika Würtenberger, David A. Groneberg, Stefanie Mache

**Affiliations:** 1 Institute for Occupational and Maritime Medicine (ZfAM), University Medical Center Hamburg-Eppendorf (UKE), Hamburg, 20459 Germany; 2 Institute of Occupational Medicine, Social Medicine and Environmental Medicine, Goethe University Frankfurt, Frankfurt, 60590 Germany

**Keywords:** Digitization, ICT, Technostress, Burnout, Job satisfaction, Primary care, Germany, Preventive measures, Multimethod

## Abstract

**Background:**

Digital technologies are increasingly being integrated into healthcare settings, including the ambulatory sector of general practitioners, with potential improvement in everyday work life. Although the changes sound very promising, the adoption of new technologies can also introduce additional stressors for medical staff, potentially resulting in negative impacts on work performance and health. This study seeks to identify the stressors and resources associated with digitization among general practitioners in Germany, explore their effects on work and health variables, and uncover potential preventive measures to mitigate these stressors.

**Methods:**

This mixed methods study combined quantitative and qualitative approaches. An online questionnaire was used to examine the relationships between technostress creators, inhibitors, and the perception of technostress, as well as the measures of burnout, job satisfaction, and general health among 114 general practitioners in Germany's ambulatory care setting. The study was carried out between March and June 2024. Several validated instruments were employed, including the Technostress Model and selected items from the Copenhagen Psychosocial Questionnaire (COPSOQ III). Exploratory assumptions were evaluated using descriptive statistics and multiple regression analyses.

**Results:**

The study found medium levels of technostress perception among the participating general practitioners (*n* = 114) along with a substantial level of technostress inhibitors. The general practitioners surveyed in this study reported experiencing burnout symptoms occasionally, expressed a moderate level of job satisfaction, and generally described their health status as good. The relationships between stressors and work- and health-related outcomes were analyzed.

**Conclusions:**

This study offers a preliminary overview of the persistence of techno-stressors, technostress inhibitors, and technostress levels and their impact on health- and work-related outcomes among general practitioners in Germany. The findings indicate that using information and communication technologies can lead to heightened stress, increased burnout symptoms, and reduced job satisfaction. As the workload for general practitioners is expected to grow in the upcoming years, the study highlights the critical need for additional preventive strategies to mitigate stress and improve well-being among general practitioners.

**Supplementary Information:**

The online version contains supplementary material available at 10.1186/s12913-025-12653-5.

## Introduction

In recent years, the healthcare landscape has undergone a profound digital transformation, changing the delivery and management of medical services. This shift has reshaped the patient experience and significantly impacted healthcare professionals' work and private lives [1, 2]. The implementation of information and communication technologies (ICTs), has emerged as a cornerstone of this transformation, promising to enhance both the working conditions of healthcare professionals [3] and the quality of care provided to patients, as well as their health behavior [4, 5]. Multiple studies have highlighted the advantages of ICT, such as enhanced communication among healthcare professionals, streamlined work processes, and improvements in documentation traceability [6–8].

Not only is the transformation in clinical settings enormous but also in the ambulatory setting, several changes are ongoing and impact the lives of healthcare professionals [9]. The implementation of electronic health records, telemedicine platforms, and mobile health applications has revolutionized the practice of primary care in various ways [10, 11]. The deployment of telemedicine offerings enables practices to reach more patients, which is particularly beneficial for rural areas and patients with limited mobility [11]. General practitioners are increasingly advocating for the use of mobile health applications for the prevention or self-management of illnesses, foreseeing that patient-focused apps will be part of their future practices [12]. In 2023, the proportion of medical practices with almost completely digitized patient documentation was 81%, with general practitioners being the most strongly represented group [9].

Although the changes sound very promising, the effects of digitalization in primary care are still contradictory for patients and general practitioners [13]. Despite all the benefits of using ICT, it can lead to stress among health professionals stemming from the high cost associated with these technologies, issues surrounding usability, and the high workload in combination with overtime work [14, 15]. Studies have also shown that the extended use of ICT results in stress for 73% of people employed in healthcare, and up to 40% experience moderate to high stress levels [16]. This experienced stress is so-called technostress [17] and can lead to core and secondary symptoms of burnout [18]. This stress was first introduced by Brod and described as “a modern disease of adaptation caused by an inability to cope with the new computer technologies healthily” [17].

Technostress is an increasing burden for general practitioners in Germany, and there is a growing workload and number of patients they treat every day [19] due to the expanding shortage of general practitioners [20]. They also experience challenges related to personnel issues, practice organization, and cooperation with other healthcare providers [21].

This leads to more experienced personal work-related burnout symptoms and lower job satisfaction among this group of healthcare professionals. The abovementioned challenges and rising burdens, such as administrative requirements, pressure to keep medical records up-to-date, and increasing work demands, lead to elevated stress levels [22].

In this way, general practitioners in Germany face various stressors in their daily work lives [21]. Therefore, it is important to generate further research in the ambulatory setting, especially in the primary care setting, to understand digital stressors further. Learning more about the interaction between relevant stressors and resources is important.

### Theoretical framework

One of the models used in this study is the Technostress model, according to Raghu-Nathan et al. Technostress is caused by the usage of ICT by end-users in organizations. The concept of technostress originated from several studies on the transactional theory of stress and coping by Lazarus et al. [23]. In the technostress model by Raghu-Nathan, the focus is on examining the so-called techno stressors (digital stressors) and the technostress inhibitors (protective factors). Researchers have reported that techno stressors can be divided into the following criteria: “techno-invasion,” “techno-overload,” “techno- complexity,” “techno-uncertainty,” and “techno-insecurity” [24].

Therefore, technostress inhibitors can mitigate the negative psychological effects, such as fear, exhaustion, and a feeling of ineffectiveness [25], induced by the stressors [24]. These measures include promoting literacy facilitation, providing technical support, and enhancing employee engagement in understanding and adapting to current digital technologies and the underlying reasons for their implementation [24]. Literacy facilitation, in particular, involves equipping employees with the knowledge and skills necessary to effectively navigate and utilize digital tools, reducing confusion and boosting confidence. By implementing technostress inhibitors, employees can better maintain a healthy lifestyle, reduce the impact of technostress on their overall welfare, and improve their digital habits [24].

The job demands-resources (JD-R) model has been used to understand the relationships between the organizational environment and employee well-being and performance. It posits that job characteristics can be categorized into two different groups: job demands and job resources [26]. Job demands are aspects that require continuous effort and are associated with physical and psychological costs. Job resources refer to aspects that facilitate achieving work-related goals and mitigate high work demands and personal growth [27]. The JD-R model helps explain burnout as an outcome of the imbalance between job demands and job resources [27]. In the literature, burnout is defined as a persistent negative mental state associated with work-life conditions in employees [28]. It is characterized primarily by exhaustion, restlessness, tension, diminished motivation, and dysfunctional work attitudes and behaviors. Our study measured the three core symptoms of burnout: emotional exhaustion, depersonalization, and reduced performance ability [28].

The job demands-resources model explains burnout and provides insights into other important work-related outcomes, such as job satisfaction. When job resources are adequate to meet job demands, employees are more likely to experience greater job satisfaction [26]. As defined by Edwin A. Locke, job satisfaction is a positive and pleasurable emotional state resulting from evaluating one's job and job experiences [29]. There is a negative correlation between job satisfaction and technostress. Technostress even leads to a reduction in commitment and the intention to stay in an organization. Technostress inhibitors, such as skills development, integration, and technical support, can counteract this [24].

### Current state of research

The issue of burnout among general practitioners has been extensively examined in the scientific literature. Numerous studies consistently highlight a high prevalence of burnout symptoms and chronic stress in this population [21, 22, 30]. Research indicates that burnout syndrome is frequently linked to substantial workloads and prolonged working hours [22]. The existing scientific research on technostress shows that healthcare workers in clinical settings experience moderate levels of technostress [31, 32]. A systematic review from 2021 revealed that in all 22 included studies, digitization in healthcare led to increased technostress among healthcare professionals [33]. Another recent study reported that healthcare specialists who regularly use electronic health records (EHRs) experience technostress, with primary care-oriented specialists being the most represented group [34].

However, no studies currently address techno-stressors concerning burnout and job satisfaction among general practitioners. This underscores the need for further research to examine the impact of technostress on the mental health and job satisfaction of general practitioners. These findings could form the basis for recommendations for reducing technostress and thus achieving better mental well-being among general practitioners.

### Objectives

This study aims to conduct a quantitative investigation to (1) better understand the relationships between the use of digital technologies and burnout, job satisfaction, and general health status. Another aim of the study is (2) to pinpoint the techno stressors and resources stemming from the utilization of digital technologies and, therefore, (3) the relationship between mental health and work-related outcomes. In addition, this study (4) examines the need for future preventive measures as well as (5) strategies for managing and coping with digital stress. These preventive measures aim to mitigate the negative literacy impacts of digital stress and promote healthier work environments for healthcare professionals.

Based on the information provided, the following assumptions have been formulated, followed by the original publication by Ragu-Nathan et al. [24].

**Hypothesis 1** Increased expression of technostress

- is positively associated with higher rates of burnout symptoms.
- is negatively associated with job satisfaction.
- is correlated overall with a lower-rated general health status.

**Hypothesis 2 **Increased presentation of technostress inhibitors

- is negatively associated with the expression of burnout symptoms.
- is positively associated with greater job satisfaction.
- is overall correlated with a higher-rated general health status.

**Hypothesis 3 **There are significant differences in the levels of technostress and the prevalence of burnout symptoms

- between different age groups of general practitioners.
- between different geographical locations of the practices: urban or rural patient care.
- between self-employed general practitioners and salaried general practitioners.

**Hypothesis 4 **Higher perceived benefits of preventive measures are associated with lower levels of technostress experienced by general practitioners.

**Fig. 1:**
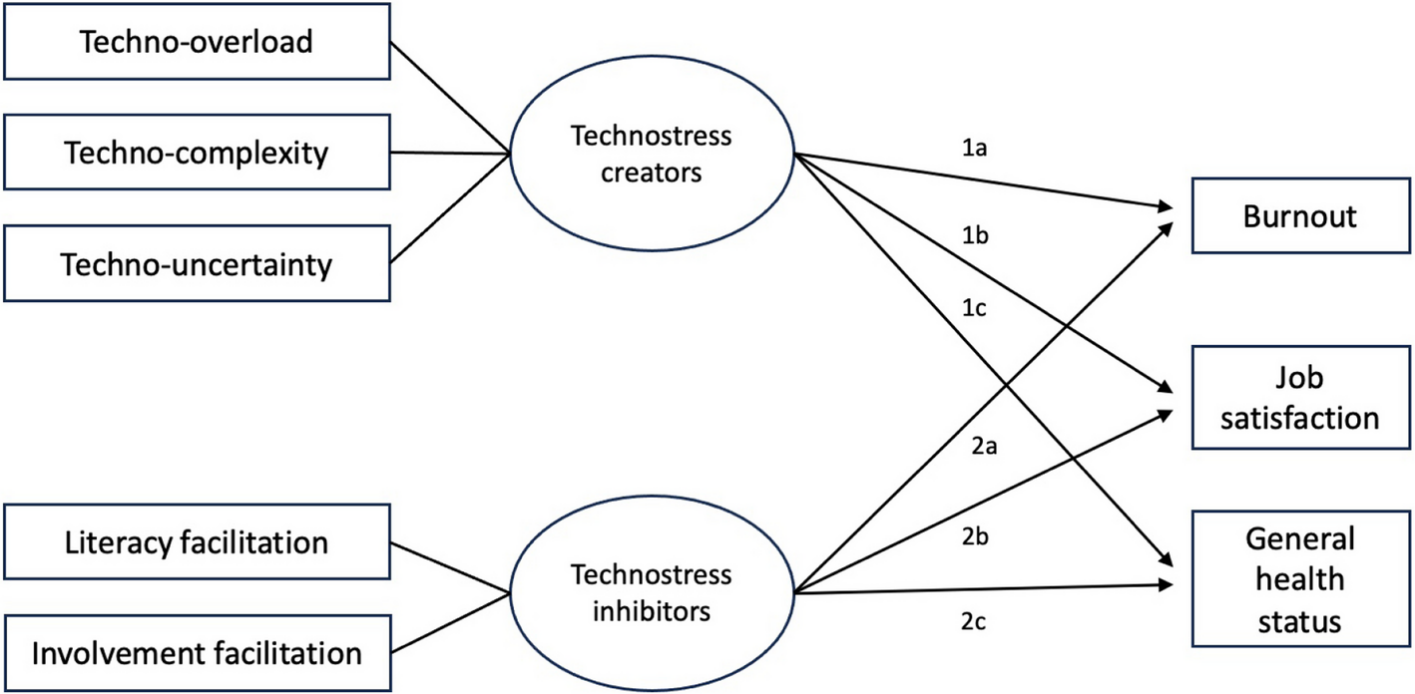
Conceptual model of the formulated hypothesis 1 and 2

A schematic representation of the conceptual model encompassing assumptions one and two is presented in Fig. 1.

## Materials and methods

### Study design and sample characteristics

This study was conducted as a cross-sectional, online-based questionnaire survey administered across multiple general practitioner offices in Germany. Working as a general practitioner with a specialization in internal medicine or general medicine was an eligibility criterion for the study participants. Further criteria were defined such that the study participants must work with digital technologies for documentation in their practices, such as EHRs or other documentation software, or additional hardware/digital devices at least once a week, meaning that the practice must have implemented at least one such technology. Multiple general practitioners working in the same practice were allowed to participate.

The size of the practice, the number of employees, and the employment status of the general practitioners (employed/self-employed) did not influence the selection process.

Our online questionnaire presented different questions to both employed and self-employed general practitioners to discern potential disparities in experienced stressors. We aimed to identify the most prevalent stressors among self-employed general practitioners and employed doctors. Additionally, we sought to compare the availability of preventive measures offered to employed doctors by their employers with those provided by self-employed general practitioners, focusing on assessing any gaps between offered and perceived provisions. An overview of the questionnaire applied in the current study can be found in an additional file.

### Data collection

The online survey was undertaken over roughly three months, from the middle of March 2024 to June 2024. The different general practitioners were identified with several different internet portals and online research. The practices were reached out to through email communication. After four weeks, a reminder to participate in the study was sent to all the general practitioners.

Before commencing the actual data collection, the online questionnaire was reviewed by three general practitioners to ensure thematic relevance and the comprehensibility of the items used. The feedback received was incorporated into the questionnaire.

### Variables and measurement

Based on the theoretical foundation, as outlined in our conceptual model, we structured our approach in which technostress creators were assessed as job demands and technostress inhibitors as job resources, based on the JD-R model [26, 27]. Technostress creators and inhibitors were included as independent variables and moderators in the model. We evaluated three outcome variables: burnout, job satisfaction, and the workload resulting from general practitioner activities.

### Sociodemographic and work-related variables

In the initial segment of the online survey, the respondents were asked to provide demographic details, including their age, gender, and the regional setting of their practice (urban, small town, rural). Additionally, participants were requested to provide information on the type of practice they were associated with, their tenure at the practice, and the extent of their professional commitments. Further data collected encompassed their cumulative professional experience, the size of their practice, and the composition of their staff, comprising both nonmedical and medical personnel. Both employed and self-employed general practitioners received the same questions in this thematic block.

### Technostress creators and inhibitors

In the second thematic section, the occurrence of digital stressors in the workplace of general practitioners is measured. For this purpose, the standardized and validated technostress scale by Ragu-Nathan et al. [24] was used in an adapted version. The three technostress creators, “techno-overload,” “techno-complexity,” and “techno-uncertainty,” were included in the German version, with a total of 10 items. The response scale ranged from 1 ('strongly disagree') to 5 ('strongly agree'), allowing for clear interpretation of scores. This inquiry demonstrates satisfactory to good reliability, as evidenced by Cronbach's alpha values ranging from 0.71 to 0.87. It exhibits strong discriminant and convergent validity, with no notable error correlations among the items [24]. In addition, for further understanding and a more specific query of the stressors, we self-developed an item based on the HIMSS study [35]. Both employed and self-employed general practitioners received the same questions in this thematic block except for the specific query of stressors.

To measure the protective factors or resources in the workplace of general practitioners, the next block of the questionnaire contained the two technostress inhibitor constructs, “literacy facilitation” and “involvement facilitation,” from the technostress scale by Ragu-Nathan et al. with a total of 6 items [24]. The response scale ranged from 1 ('strongly disagree') to 5 ('strongly agree'). In this section, both employed and self-employed general practitioners were given identical questions, apart from the inquiry regarding the construct of “involvement facilitation”, which refers to creating opportunities for employees to engage in decision-making processes actively and giving them a sense of control and participation in the integration of digital tools into their work environments.

### Preventive measures

In the third thematic part, numerous items were introduced to evaluate the preventive measures already implemented in the practice, focusing on two key constructs: the effectiveness and the perceived benefit of these measures. The preventive measures were assessed using a Likert scale with seven items [36, 37]. The Likert scale ranged from 1 ('Do not agree') to 5 ('Agree'). For a more detailed insight into already implemented preventive factors, another self-developed section in the examination was included with a total of four items. These items focused on identifying specific supplementary measures that the practice has put in place. These items were complemented by a self-developed scale querying the advantage of the already implemented preventive measures. The specific preventive measures addressed in these items included the availability of sufficient devices, the stability of the system, the use of devices that do not interfere with doctor-patient communication, and the implementation of new digital technologies only if reliable remote maintenance can be ensured. Additionally, three supplementary questions in open-text format were included to capture both the positive and negative aspects of these measures, as well as to identify any perceived need for additional preventive measures. These open-ended responses provided a deeper understanding of the practitioners’ perspectives. The self-employed general practitioners were also asked what specific support they hoped to receive from official bodies such as health insurance companies, political authorities, or other relevant organizations to manage digital stress. In this section, employed and self-employed general practitioners were given different items to answer for a deeper understanding of how preventive measures differ in their perceived effectiveness by self-employed and employed general practitioners.

### Work-related, mental, and general health outcomes

In the final segment of the questionnaire, various health- and work-related outcomes were scrutinized using standardized and validated measures. The extent of burnout symptoms experienced by general practitioners was evaluated using self-designed items inspired by the core symptoms outlined in the Maslach Burnout Inventory and supplemented by scales adapted from the validated COPSOQ framework [36, 38]. The response scale for burnout symptoms ranged from 1 ("Never/rarely") to 5 ("Always"). Additionally, job satisfaction and the extent of workload arising from general practitioner activities were evaluated using questions from the IHP Survey [39]. Job satisfaction was measured on a 5-point scale ranging from 1 ("Not satisfied at all") to 5 ("Extremely satisfied"), while workload perception was assessed on a 5-point scale ranging from 1 ("Not strenuous at all") to 5 ("Extremely strenuous"). The validity and reliability of the COPSOQ framework have been well-established in previous research, with Cronbach's alpha values indicating strong internal consistency for its scales [36]. Additionally, job satisfaction and the extent of workload arising from general practitioner activities were evaluated using questions from the IHP Survey, which also provides validated and reliable data. The IHP Survey employs a randomized sampling method to ensure representativeness and uses standardized data collection procedures, such as telephone interviews, to enhance reliability [39]. The overall subjective health status was measured using a 10-point scale ranging from 0 (indicating the lowest level of health) to 10 (reflecting the highest level of health), as outlined in the COPSOQ [36]. The COPSOQ scale exhibited strong reliability, indicated by a Cronbach's alpha value of 0.84 [36].

In total, six items were included in this section of the questionnaire, with both employed and self-employed general practitioners receiving identical questions.

### Statistical data analysis

Statistical analyses were performed using IBM SPSS software, version 29.0.2.0. The dataset was rigorously examined for plausibility, and any missing values were systematically addressed. Missing data for individual responses were imputed using appropriate data imputation methods to maintain the robustness of the analysis. We used 95% confidence intervals and an alpha level of ≤ 0,05 for significance tests. For correlation analyses, Spearman’s Rho correlation coefficient for ordinal variables and Pearsons’s correlation coefficient for continuous variables. Furthermore, we incorporated multiple regression analyses controlling for extraneous variables. Parametric test procedures (T-test, ANOVA) were used after evaluating for normal distribution of the variable data. In addition to the non-parametric tests, the Mann–Whitney U test and the Kruskal–Wallis test were employed to analyze group differences.

For the qualitative analysis, an inductive content analysis was conducted to systematically evaluate the three open-text formats. The responses were coded and categorized following the principles of qualitative content analysis. Thematic patterns were identified, and key themes were extracted to provide insights into participants' subjective experiences and perspectives. The qualitative findings were then triangulated with quantitative results to enrich the interpretation of the data.

## Results

### Sample description

A total of 114 general practitioners working in the ambulatory setting of general practitioners participated in the online survey. Most of the participating physicians were female (53.5%, *n* = 114). Additionally, 47.3% of the general practitioners were aged between 55–64 years, and 49% had over 25 years of professional experience. Most participants (93%) were self-employed, with 93.9% working full-time (35 h or more per week). Moreover, 60% worked in solo practices, predominantly located in urban areas with populations exceeding 50,000 (54%), and 50.9% had 2- 5 medical professionals employed in these practices. The median number of patients treated per week at the participating practices was between 200 and 300. (See Table 1 for detailed demographics).

**Table 1:** Characteristics of the study population and practices (*n* = 114)

Characteristics	Frequency (n)	Percentage (%)
*Gender*		
Male	53	46.5%
Female	61	53.5%
*Age*		
35–44 years	19	16.7%
45–54 years	32	28.1%
55–64 years	55	48.2%
> 65 years	8	7%
*Job position*		
Self-employed physician	106	93%
Employed physician	8	7%
*Extent of current working hours*		
Working full time (≥ 35 h/week)	107	93.9%
Working part time (15–34 h/ week)	7	6.1%
*Practice type*		
Solo practice	64	56.1%
Group practice	43	37.7%
Multidisciplinary group practice	3	2.6%
Multidisciplinary medical care center	4	3.5%
*Location of practice*		
Rural (fewer than 5,000 inhabitants)	62	54.4%
Small town (5,000–50,000 inhabitants)	36	31.6%
Urban (> 50.000)	16	14%
*Patients treated per week per practice*		
< 100	7	6.1%
100–200	31	27.2%
200–300	31	27.2%
300–400	22	19.3%
400–500	7	6.2%
> 500	16	14%

Regarding the usage of digital documentation technologies, electronic health records (EHR) were the most frequently chosen option, with 96% of the general practitioners selecting it. This was followed by the usage of additional software and digital services (74%), and the usage of additional hardware (73.7%).

### Descriptive statistical analysis

The average technostress level among all participants was measured at a medium level, with a mean score for the three technostress creators of M = 3.01 and a standard deviation of 1.43. Among the individual technostress creators, the highest mean was observed for techno-uncertainty (M = 3.42, SD = 1.44), followed by techno-overload (M = 3.3, SD = 1.38) and techno-complexity (M = 2.43, SD = 1.29).

Additionally, several potentially harmful side effects and stress-inducing elements have been investigated. The most frequently reported stressor among self-employed general practitioners was the high cost of maintenance and updates, with 51.9% stating that they feel very often stressed by this issue. Other significant stressors included technical errors in the system and the health insurance companies' control tools, with 38.7% of the respondents feeling very stressed by these factors. Another major issue was the increase in time expenditure, with 55.7% of self-employed participants reporting that they often or very often feel stressed by this problem, resulting in less time available for their employees. However, double documentation did not appear to be a significant concern for the self-employed general practitioners.

For employed general practitioners, the most stress-inducing aspects were technical errors in the system and an increase in time expenditure, with 50% of respondents reporting that they often feel stressed by each of these issues.

### Technostress inhibitors and resources

The overall expression of technostress inhibitors among participants indicated a substantial level of persistent resources, with a mean score of M = 3.83 (SD = 1.17). Specifically, literacy facilitation had an average score of M = 3.67 (SD = 1.16). Involvement facilitation, however, had a higher mean score of M = 4.13 (SD = 1.12), suggesting that participants experienced a significant level of engagement and support in mitigating the effects of technostress.

### Preventive measures in the use of digital technologies

Preventive measures and actions were generally applied very frequently by self-employed general practitioners, with an average score of M = 3.77 (SD = 1.05). The most used preventive measure was information, with practitioners reporting that they inform their employees in a timely and sufficient manner before the implementation of new technologies, with a mean score of M = 4.08 (SD = 0.88). However, participation remains a preventive measure that is not sufficiently implemented. Overall, 71.8% of employed general practitioners reported that they are not significantly involved in finding technical solutions, selecting new products, or evaluating new products.

Furthermore, 94.7% of the participating general practitioners reported sufficient provision of technical devices. However, 60.5% indicated that ensuring system stability before implementation to avoid time-consuming double documentation is not consistently practiced. Additionally, 62.3% mentioned that the use of devices that do not hinder doctor-patient conversations is not adequately implemented. Moreover, 45.6% stated that the introduction of new digital technology should only occur when reliable remote maintenance can be ensured, but this condition is not always met. Overall, the average level of satisfaction with the preventive measures implemented thus so far was moderate (M = 3.13, SD = 1.14), indicating a mixed but generally satisfactory response.

### Work-and mental health-related outcomes

Overall, the persistence of burnout symptoms among general practitioners was moderate, with an average score of M = 3.03 (SD = 1.03). These findings indicate that general practitioners occasionally experience burnout symptoms. On average, most general practitioners reported being reasonably satisfied with their job (M = 3.06, SD = 1.14) and found their job to be somewhat too very strenuous (M = 3.69, SD = 0.9). In terms of their perceived general health status, the participating general practitioners rated their health as good, with an average score of M = 7.01 (SD = 1.64). While the standard deviation was relatively high, the responses ranged from 1 to 10 on the scale.

### Qualitative data analysis

In addition to the previously described quantitative findings, the participating general practitioners provided detailed insights into the specific aspects of the digital documentation technologies they found to be stress-inducing. They also shared strategies to reduce technostress in their practices and information on the preventive measures they plan to implement in the future. Furthermore, they outlined the types of support they would need from health insurance companies, political entities, and other relevant organizations to manage technostress effectively. Four primary categories and 18 subcategories were identified (see overview table), which are elaborated upon in the following sections (Table 2).

**Table 2:** Qualitative evaluation of items with free-text answers in the form of a category system

Items/Main categories	Subcategories
Stress-inducing aspects	▪ technical issues/underdeveloped technical solutions ▪ inadequate adaptation of usage to daily practice ▪ insufficient support from IT companies
Technostress reducing strategies	▪ Hiring trained personnel/IT specialists ▪ Team meetings/discussions to find solutions ▪ Selecting software providers with strong support ▪ Early engagement with hardware and software updates ▪ Time limitations/reduction of office hours
Future preventive strategies	• Staff training • Guidelines for the use of digital technology • Alternative simpler digital software programs • More time on documentation therefore less time for patient care • No actions due to time constraints, economic situation, or staff shortages
Support from health insurance companies, political entities, and other relevant organizations	• Financial relief and subsidies for introducing new technology, especially amidst increasing IT costs. • Implementation of thoroughly tested and stable systems, ensuring uniformity. • Reduction of bureaucracy and streamlined documentation. • Centralized support for questions and issues, assistance during implementation.

#### Stress-inducing aspects of digital documentation technologies

The participating general practitioners were initially surveyed to gather insights into the stress-inducing factors associated with digital documentation technologies. Out of 114 general practitioners, 38 responded to this inquiry. The primary stress sources were technical challenges, insufficiently developed technological solutions, and a lack of integration into daily clinical workflows. These factors collectively contributed to significant burdens within their practices. Specifically, regularly occurring technical system failures and continuous updates, were perceived as stress-inducing because they often led to longer working hours and increased workload. In addition, insufficient support from IT companies was called stressful, as it often led to long waiting hours in support hotlines and additional costs for the practice.

*“With more options, everything becomes increasingly complex. Since paperless patient administration systems (PVSs) were implemented, I have worked 1- 2 h longer each day. However, the improvement in patient care does not seem to justify the additional time spent." *(P139; Engl. Translation of original citations).

#### Technostress-reducing strategies

When asked about the strategies to overcome technostress in general medicine, a total of 61 participants provided further insight. Hiring trained personnel or IT specialists is a crucial step, as it ensures that technical issues can be swiftly and effectively managed, reducing the number of general practitioners. Regular team meetings and discussions are also essential, providing a forum for sharing experiences, brainstorming solutions, and fostering a collaborative environment. The selection of software providers with strong support and early engagement with hardware and software updates were also referred to as stress-reducing strategies.

#### Future preventive strategies

Research on future preventive strategies to reduce technostress among general practitioners is a key area of interest. A total of 37 general practitioners out of 114 responded to this query. One of the primary strategies they plan to implement is comprehensive staff training programs to increase proficiency and comfort with digital tools. Additionally, they aim to introduce clear guidelines for the effective use of digital technology. Exploring and adopting alternative, simpler digital software programs is also seen as a beneficial approach. However, while they plan to allocate more time for digital documentation, this may inadvertently reduce the time available for direct patient care. Despite these plans, certain constraints, such as limited time, economic challenges, and staff shortages, may hinder the implementation of these measures, emphasizing the need for a balanced and feasible approach to digital integration.

##### Support from health insurance companies, political entities, and other relevant organizations

Moreover, when queried about their desire for support from health insurance companies, political entities, and other relevant organizations, 74 general practitioners responded. A primary concern expressed was the need for financial relief and subsidies to offset the rising costs of IT implementation.

*“The practices are forced to implement digitalization and must spend money on hardware, software, and IT support. Despite this, these investments represent an additional time and financial burden owing to malfunctions, expired certificates, connector replacements, and other issues”* (P10; Engl. Translation of original citations).

Another significant request was to adopt rigorously tested and stable systems to promote consistency, streamline documentation, and reduce bureaucratic hurdles. Additionally, there was a strong desire for centralized support services to address queries and assist with the implementation of new technologies.

### Analytical statistical analysis

The analysis of our first hypothesis revealed a mild positive correlation for the variables of technostress and burnout *r* = 0.36 (CI: 0.19, 0.51) and a slight negative correlation between technostress and job satisfaction *r* = −0.34 (CI: −0.49, −0.16 ) with *p* < 0.01, supporting hypotheses 1a and 1b. For the variables technostress and the general health status, our analysis revealed a modest negative correlation *r* = −0.16 (CI: −0.34, 0.02), which was not significant (*p* > 0.05). For this reason, hypothesis H1c cannot be confirmed (Table 3).

**Table 3:** Correlation for Hypothesis 1

Hypothesis	Pearson’s r	95% CI	*p*-value
**H1a**	0.36	[0.19, 0.51]	<0.01
**H1b**	−0.34	[−0.49, −0.16]	<0.01
**H1c**	−0.16	[−0.34, 0.02]	>0.05

The multiple regression analysis of the technostress creator variables and burnout outcomes revealed that the three independent variables, techno-overload, techno-complexity, and techno-uncertainty, accounted for 14.7% of the variance in burnout within this model. These results were significant (*p* < 0,001). The influence of the predictor techno-uncertainty was slightly significant (*p* < 0.05). However, for the other predictors, the findings were not significant (*p* > 0.05). The analysis of the outcomes of job satisfaction and general health status revealed no significant associations (Table 4).

**Table 4:** Multiple regression analysis of techno-overload, -complexity, -uncertainty, and the outcome variables of burnout, job satisfaction, and general health status

Predictors	B	SE	t	*p*
**Outcome of burnout**				
Techno-overload	0.150	0.082	1.831	0.07
Techno-complexity	0.064	0.087	0.736	0.436
Techno-uncertainty	0.259	0.103	2.502	0.014
Notation *R*2=0.147 (*n*=114, *p*<0.001)				
**Outcome of job burnout**				
Techno-overload	−0.297	0.108	−2.745	0.007
Techno-complexity	−0.35	0.115	−0.302	0.763
Techno-uncertainty	−0.128	0.137	−0.934	0.352
Notation *R*2=0.108 (*n*=114, *p*>0.05)				
**Outcome of General health status**				
Techno-overload	−0.369	0.176	−2.100	0.038
Techno-complexity	0.046	0.187	0.247	0.805
Techno-uncertainty	0.01	0.223	0.046	0.963
Notation *R*2=0.0142 (*n*=114, *p*>0.05)				

Correlation analyses between technostress inhibitors and burnout revealed a nonsignificant negative correlation (*r* = −0.04, *p* > 0.05). Additionally, the analyses examining the relationships between technostress inhibitors and job satisfaction and general health status showed non-significant associations (*p* > 0.05). Thus, hypotheses 2a, b, and c cannot be verified (Table 5).

**Table 5:** Correlation for Hypothesis 2

Hypothesis	Pearson’s r	95% CI	*p*-value
**H2a**	−0.04	[−0.222, 0.145]	*p*>0.05
**H2b**	0.047	[−0.138, 0.229]	*p*>0.05
**H2c**	0.029	[−0.156, 0.212]	*p*>0.05

To test Hypothesis 3a, we conducted a one-way ANOVA to assess the influence of age on technostress levels. However, the level of technostress did not differ statistically significantly among the different age groups with F (df: 3, 110) = 2.578, *p* > 0.05. In addition, we further conducted a Kruskal–Wallis analysis to identify whether the expressions of burnout symptoms differed among the different age groups. However, our findings were insignificant, meaning there were no differences between the various age groups.

To test Hypothesis 3b, we conducted a one-way ANOVA to compare technostress levels across different geographical locations. The analysis (F (df: 3, 110) = 2.578, *p* > 0.05) yielded nonsignificant results, leading us to retain the null hypothesis. This finding was further supported by eta-square values of 1.401 between groups and 59.842 within groups, suggesting that technostress levels do not significantly differ between urban and rural patient care settings. Similarly, the results of the Kruskal–Wallis test (*p* = 0.355) confirmed the absence of significant differences in burnout symptoms across geographical locations. Additionally, in testing Hypothesis 3c, neither the t-test for independent samples (*p* > 0.05) nor the Mann–Whitney U test (*p* > 0.05) produced significant results, suggesting that technostress levels and the prevalence of burnout symptoms are quite similar between self-employed and salaried general practitioners in our study population (Table 6).

**Table 6:** Influence of demographic variables on technostress

Hypothesis	Test conducted	F (df)	*p*-value
H3a	One-way ANOVA	2.578 (3,110)	>0.05
H3b	One-way ANOVA	2.578 (3,110)	>0.05
H3c	t-test & Mann-Whitney U	-	>0.05

To test Hypothesis 4, we conducted a one-way ANOVA to compare technostress levels with the perceived benefits of preventive measures. The analysis revealed a statistically significant difference in technostress levels across the perceived benefit categories (F (df: 3, 110) = 6.536, *p* < 0.001). This finding was further supported by eta-squared values of 11.848 between groups and 49.396 within groups, indicating a meaningful variation in technostress levels based on perceived benefits. To further explore these differences, we performed a post-hoc analysis to identify which groups differed. The Tukey HSD test indicated significant differences between the "low" and "very high" groups, with a mean difference of −1.02 (*p* < 0.05), between the "satisfying" and "very high" groups, with a mean difference of −0.62 (*p* < 0.001), and between the "low" and "high" groups, with a mean difference of −0.7 (*p* < 0.05). Thus, Hypothesis 4 can be verified (Table 7).

**Table 7:** Results for technostress and perceived benefits

Hypothesis	Test conducted	F (df)	*p*-value
H4	One-way ANOVA	6.536 (3,110)	<0.001

## Discussion

This is the first study focused on exploring the digital stressors and resources affecting German general practitioners. Our goals were to understand these stressors better, identify preventive strategies to reduce technostress and investigate the potential connections between health and work-related outcomes. Additionally, we aimed to identify preventive measures to minimize technostress.

### Key-results

The data from our study indicate that the surveyed general practitioners experienced a moderate level of technostress, with techno-uncertainty emerging as the most significant factor. This stressor is closely linked to general practitioners’ concerns about the high costs of system maintenance and updates, the oversight exerted by health insurance companies through control tools, and the occurrence of technical errors within digital systems. These findings are consistent with recent studies in the broader medical sector, which have also reported moderate levels of technostress among physicians in Germany and Switzerland [13, 31, 32, 40]. However, unlike our study, those studies identified techno-overload as the primary source of technostress. This suggests that the unique characteristics of ambulatory care, such as its decentralized nature and specific patient interaction demands, shape the technostress experienced by practitioners in this setting. It is important to cautiously approach comparisons with literature, as no previous studies have specifically examined digital stress within the ambulatory care sector.

A detailed analysis of the relationships between technostress and various health- and work-related outcomes, our results revealed a significant positive correlation between technostress and burnout, as well as a significant negative correlation between technostress and job satisfaction. Additionally, we observed a slight negative, however not significant, correlation between technostress and the subjectively perceived general health status. These results are in line with the recent literature. A study conducted in the United States found a significant association between the use of electronic health records (EHR) and increased burnout risk among primary care physicians [15]. Another systematic review examining the relationship between various mental health and work-related outcomess in different sectors found that technostress is negatively associated with job satisfaction and positively associated with burnout [41]. Other recent quantitative studies in the medical field have also shown a significant positive correlation between technostress creators and burnout symptoms, as well as significant negative correlations between the variable of technostress and general health status [13, 31]. However, technostress should not be regarded exclusively as a negative phenomenon. Emerging research, particularly the techno-eustress and distress model by Califf et al. suggests that while technostress has a substantial impact on job satisfaction, it also allows for positive effects by fostering improved performance, job satisfaction, and personal development under certain conditions [42].

Our study revealed elevated scores among the polled techno inhibitors, literacy facilitation, and involvement facilitation. One possible reason for this might be that most of the participating general practitioners were self-employed, requiring them to increase participation in IT processes and self-managing their software programs. This increased involvement in technology could impact their technology self-efficacy, and therefore reduce technostress [43]. The associations between the use of technostress inhibitors with the measures of burnout, job satisfaction, and general health status were either not significant or very low. Recent German clinical studies have found little to no significant link between technostress, the use of technostress inhibitors, and the outcomes of burnout, job satisfaction, or overall health [31, 32, 40]. Another recent study showed that technostress inhibitors had mediating effects on perceived technostress levels and employers’ well-being [44].

The general practitioners surveyed in this study reported experiencing burnout symptoms occasionally, expressed a moderate level of job satisfaction, and reported that their work was somewhat strenuous. Despite these challenges, they generally described their health status as good. The prevalence of burnout symptoms observed in our study aligns with other published German research [22, 30], indicating that burnout symptoms in general practice are common but not as severe as among physicians in other medical specialties [45]. Studies from across Europe have highlighted that general practitioners frequently experience burnout, with some study populations experiencing even higher rates of burnout symptoms than our population does [46–48]. Furthermore, a 2022 systematic review examining the global prevalence of burnout among general practitioners confirmed that burnout is a widespread concern within this professional group worldwide [49].

When we compared the level of job satisfaction in our study population with other German studies, we observed lower levels of job satisfaction [22]. A potential explanation for this discrepancy could be that the study by Werdecker et al. [22] collected data in 2018, before the onset of the COVID-19 pandemic. Since then, the workload for general practitioners has significantly increased, likely contributing to heightened frustration and reduced job satisfaction [50, 51].

Despite these challenges, the general practitioners in our study rated their health status as good, a finding that is consistent with other European literature [52] and similar studies across other medical specialties [31].

When we analyzed the potential differences in the two measures of technostress and burnout symptoms between the different age groups, we did not find statistically significant differences. In a German study, that estimated burnout, satisfaction, and happiness among general practitioners, age was negatively associated with personal and work-related outcomes [22]. Additionally, in a French study, general practitioners were at high risk of burnout, especially those over 50 years old who were significantly more affected by severe burnout symptoms [46]. A potential explanation for these differences could be the limited size of our study population. Additionally, we did not find statistically significant differences in technostress levels or burnout symptoms across various geographical locations or employment statuses among the general practitioners. These results diverge from findings in other German studies that have reported notable variations. For example, Steinhauser et al. identified significant differences in average working hours between urban and rural practices, despite similar patient volumes per week [53]. Similarly, Hansen et al. reported a greater workload for rural general practitioners [54]. Furthermore, another German study reported a greater prevalence of burnout among employed physicians compared to their self-employed counterparts [22]. A potential explanation for the discrepancies observed in our study could be the limited sample size of employed general practitioners, who composed only 7% of our study population and only 14% of the general practitioners working in rural areas (see limitations).

The qualitative analysis provided deeper insights into the preventive measures that have already been implemented, such as hiring trained personnel or IT specialists, conducting regular team meetings, and selecting software providers with robust support services. General practitioners also reported having to reduce consultation hours due to the growing complexity of digital systems, which has, in turn, increased their workload. They further shared plans to implement additional preventive measures in the future, including staff training, guidelines for the use of digital technology, and the exploration of alternative, simpler digital software solutions. As previously discussed, the COVID-19 pandemic exacerbated the workload among general practitioners [50, 51], a challenge further intensified by the growing shortage of general practitioners in Germany [20]. In 2023 alone, over 900 general practice positions remained unfilled in the federal state of Baden-Württemberg [55]. This underscores the urgent need for additional preventive measures to alleviate (techno)stress. Among the 74 general practitioners who responded to the open text field for extra support from health insurance companies, political entities, and other relevant organizations, the primary concern was the need for financial relief and subsidies to offset the rising costs of IT implementation. This concern aligns with the existing literature in Germany, highlighting the digitalization of medical practices as both cost- and time-intensive [56]. The significant increase in operating costs in recent years, particularly in personnel expenses and maintenance [57], further explains the demand for financial support. In addition to these findings, our study revealed that general practitioners who perceive greater benefits from preventive measures tend to experience lower levels of technostress. This suggests that both the preventive measures already in place and those planned may effectively reduce technostress among general practitioners.

### Strengths and limitations

The use of several validated and well-recognized scales, including the technostress scale by Ragu-Nathan et al. [24] and different scales from the COPSOQ is a strength of this study and ensures the quality of the measurements. Another strength of this study lies in the recruitment strategy, which included general practitioners from every federal state in Germany and from rural and urban areas throughout Germany. However, certain limitations of our study also need to be addressed. Owing to the small number of study participants or the underrepresentation of employed general practitioners in the study sample, it was not possible to find statistically significant differences in technostress levels among the different groups. Therefore, the recruitment strategy for future surveys should be improved to include more general practitioners, especially employed practitioners. In addition, the response rate of 9.4% (142 out of 1549 surveys) is compared to other online questionnaires quite low [13, 40]. This can be attributed to the recruitment strategy using a generic email address, which is often overlooked by doctors and read primarily by their medical assistants. Additionally, owing to the high workload of general practitioners, there might be no additional time to respond to the online survey. As noted in previous studies, low response rates are a common issue in research involving general practitioners, as structural barriers such as time constraints and limited resources frequently hinder participation [58]. Consequently, our study results must be interpreted with caution due to the small sample size, which may limit the representativeness of the results. Furthermore, the study may be subject to selection bias, as the relatively low response rate and underrepresentation of employed general practitioners may have skewed the sample, potentially limiting the generalizability of the findings to the broader population of general practitioners in Germany.

### Implications for further research

The digital transformation of patient care will continue to advance rapidly in the coming years, both in hospitals and ambulatory healthcare settings. Further investigation is especially crucial in the ambulatory sector, where a gap in the literature exists concerning comparative studies on technology use and digital stress. We encourage future researchers to conduct a longitudinal study design to be able to monitor the evolution of technostress levels and potential changes over a longer period, as the ambulatory sector is currently undergoing significant changes. In addition, future studies could evaluate the construct of technostress in a more differentiated way and investigate the positive side of the construct of technostress. The circumstances under which technostress creators can lead to eustress or distress in medical care based on the studies by Califf et al. could be analyzed [42]. By conducting comparative studies in the ambulatory sector of medical care, researchers can gain a deeper understanding of how digital stressors are perceived across different medical specialties, ultimately leading to the development of targeted strategies to mitigate digital stress and improve the overall well-being of the medical staff in ambulatory care. Future research on these variables might consider investigating additional mediating or influencing factors of technostress and the multiple outcomes, such as digital competence, self-efficacy, and back-office support.

### Practical implications

Based on the feedback from the physicians taking part in our study, we formulated recommendations, which are divided into technological, organizational, and policy aspects, to reduce and better manage technostress for the general practitioners working in the ambulatory sector of patient care. First, the careful selection of user-friendly and intuitive software programs plays a crucial role in minimizing technostress among general practitioners. As their workload continues to expand, implementing software that is designed with simplicity and ease of use in mind, general practitioners can navigate their daily tasks more efficiently and reduce their cognitive load. Additionally, technology should be tailored to general practitioners' specific needs, workplace environment, and personal engagement to increase job satisfaction [59]. General practitioners could significantly benefit from strategies that integrate extra software functionalities, such as clinical support tools and referral frameworks [60].

In addition, guidelines for using digital technology in daily practice and regularly occurring team meetings to discuss current technical issues and their improvement encourage teamwork and strengthen collaboration among team members to enhance overall productivity and effectiveness. Furthermore, general practitioners should select reliable IT support with good availability and response time to report and solve technical problems quickly. Moreover, recruiting skilled personnel, including IT specialists, and strategically distributing tasks among practice employees can significantly alleviate the individual workload and task volume associated with digital transformation in general practice. This approach ensures that responsibilities are shared more efficiently, reducing the burden on individual practitioners. At the policy level, general practitioners would benefit from measures to reduce bureaucratic hurdles and streamline documentation processes. Simplifying administrative tasks would mitigate technostress and enhance overall job satisfaction. Additionally, financial incentives, such as subsidies or tax relief for adopting new technologies, would provide crucial support [60]. These measures would help reduce the financial strain on practices, allowing practitioners to focus more on patient care and less on administrative challenges.

### Conclusion

This study provided a first overview of the persistence of techno-stressors, technostress inhibitors, and technostress levels, as well as their possible influence on relevant health- and work-related outcomes among a group of general practitioners working in the ambulatory sector in Germany. One of the main conclusions of this study is that the use of information and communication technologies can lead to increased stress and burnout symptoms among the surveyed physicians. However, despite all the ongoing challenges in general practice, the perceived technostress level among physicians is moderate. Individual coping strategies and implemented preventive measures might influence this situation. Our study underscores the urgent need to implement additional preventive measures to minimize (techno-)stress, particularly as the workload for general practitioners tends to expand over the next few years. Furthermore, there is a need for more in-depth research to better understand these challenges within this medical sector. Comprehensive research will provide valuable insights into effective interventions and best practices for managing (techno-)stress, ultimately contributing to improved well-being and job satisfaction among general practitioners.

## Supplementary Information

Additional file 1. Contents of the online questionnaire.

## Data Availability

The datasets analyzed during the current study are not publicly available due to German national data protection regulations. The datasets used and analyzed during the current study are available from the corresponding author upon reasonable request.
