# Supplementary material for: Digital stress perception and associations with work- and health-related outcomes among general practitioners in Germany: a quantitative study
Source: BMC Health Serv Res. 2025 Apr 11;25:535. doi: 10.1186/s12913-025-12653-5 (PMC11987191; doi:10.1186/s12913-025-12653-5)
Supplement: Supplementary file 1 — Additional file 1. Contents of the online questionnaire. [file 12913_2025_12653_MOESM1_ESM.docx]

**Additional File 1**

##### Overview of the questionnaire applied in the current study

| **Section** | **Items/ Scales/ subscales** | **Number of items** |
| --- | --- | --- |
| Inclusion criteria | Being a general practitioner specializing in internal medicine or general medicine, Employment at least part-time (20 hours per week), Use of digital technology (digital documentation software, digital appointment calendar, etc.) | 3 |
| Participant and hospital characteristics ^a^ | Age, Gender, Regional structure of the practice (urban, small-town, rural), Type of practice, Duration of employment in the practice, Scope of employment, Total professional experience, Patient volume per week, Number of non-medical staff working in the practice, Number of medical staff working in the practice | 10 |
| Digital stressors in the workplace (technostress)^b,c^ | *Techno-overload* ^b^  Likert scale: 1 ('strongly disagree') to 5 ('strongly agree') | 4 |
|  | *Techno-complexity* ^b^  Likert scale: 1 ('strongly disagree') to 5 ('strongly agree') | 4 |
|  | *Techno-uncertainty* ^b^  Likert scale: 1 ('strongly disagree') to 5 ('strongly agree') | 2 |
|  | *Specific stressor assessment* ^c^ | 10 |
| Stress-reducing factors/resources in the workplace (technostress-inhibitors) ^b^ | *Literacy facilitation*  Likert scale: 1 ('strongly disagree') to 5 ('strongly agree') | 4 |
|  | *Involvement facilitation*  Likert scale: 1 ('strongly disagree') to 5 ('strongly agree') | 2 |
| Preventive measures | *Information, qualification, participation*^d^  Likert scale: 1 ('Do not agree') to 5 ('Agree') | 7 |
|  | *Detailed inquiry on preventive measures*^c^  Likert scale: 1 ('Do not agree') to 5 ('Agree') | 4 |
|  | *Inquiry on the effectiveness of implemented measures*  Likert scale: 1 ('Do not agree') to 5 ('Agree') | 1 |
|  | *Inquiry on suggestions/requests for improvement*  Open text format | 3 |
| Work- and health related outcomes | *Burnout symptoms* ^a,e^  Likert scale: 1 ("Never/rarely") to 5 ("Always"). | 3 |
|  | *Job satisfaction* ^a, e^  Likert scale: 1 ("Not satisfied at all") to 5 ("Extremely satisfied"), | 1 |
|  | *Workload from general practice activities* ^a, e^  Likert scale: 1 ("Not strenuous at all") to 5 ("Extremely strenuous") | 1 |
|  | *General subjective health status* ^e^  Likert scale: 0 (indicating the lowest level of health) to 10 (reflecting the highest level of health) | 1 |
| Total number of items for each participant | | 60 |

^a^ According to Ochmann R, an der Heiden I, Bernhard J. *IHP-Survey Deutschland 2022: Befragung der Hausärztinnen und Hausärzte in Deutschland.* Berlin: IGES Institut; 2023 Apr.

^b^ According to Ragu-Nathan TS, Tarafdar M, Ragu-Nathan BS, Tu Q. The Consequences of Technostress for End Users in Organizations: Conceptual Development and Empirical Validation. *Information Systems Research.* 2008 Dec;19(4):417–33. DOI:10.1287/isre.1070.0165

^c^ According to Healthcare Information and Management Systems Society (HIMSS). *Auf den Spuren der Zeitdiebe im Krankenhaus: Die wahre Belastung durch Dokumentation an deutschen Akutkrankenhäusern wird unterschätzt*. Berlin, Germany; 2015.

^d^ According to Bräutigam C, Enste P, Evans M, Hilbert J, Merkel S, Öz F. Digitalisierung im

Krankenhaus: Mehr Technik - bessere Arbeit? [Internet]. Düsseldorf: Hans-Böckler-Stiftung;

2017 [cited 2024 Aug 18]. Available from: https://hdl.handle.net/10419/173275.

^e^ According to Lincke HJ, Vomstein M, Lindner A, Nolle I, Häberle N, Haug A, et al. COPSOQ III in

Germany: validation of a standard instrument to measure psychosocial factors at work. *Journal*

*of Occupational Medicine and Toxicology.* 2021 Dec 16;16(1):50. DOI: 10.1186/s12995-021-

00331-1
